# Physical activity intervention for the prevention of neurological diseases

**DOI:** 10.1002/hsr2.1524

**Published:** 2023-08-22

**Authors:** Burhan Kantawala, Nagham Ramadan, Youmna Hassan, Violette Fawaz, Nadine Mugisha, Abubakar Nazir, Magda Wojtara, Olivier Uwishema

**Affiliations:** ^1^ Oli Health Magazine Organization, Research and Education Kigali Rwanda; ^2^ Faculty of Medicine Yerevan State Medical University Yerevan Armenia; ^3^ Faculty of Medicine Beirut Arab University Beirut Lebanon; ^4^ Faculty of Medicine and Surgery Ahfad University for Women Omdurman Khartoum Sudan; ^5^ Faculty of Pharmacy Beirut Arab University Beirut Lebanon; ^6^ Faculty of Global Surgery University of Global Health Equity Kigali Gasabo Rwanda; ^7^ Department of Medicine King Edward Medical University Lahore Pakistan; ^8^ Department of Medicine University of Michigan Medical School Ann Arbor Michigan USA; ^9^ Department of Medicine Clinton Global Initiative University New York USA; ^10^ Faculty of Medicine Karadeniz Technical University Trabzon Turkey

**Keywords:** disease prevention, exercise, mental health, neurological diseases, physical activity

## 1 INTRODUCTION

The brain is a vital organ responsible for motor and sensory functions as well as complex processes like perception, learning, and emotions. Maintaining brain health is crucial, and it can be achieved through the increasing physical activity, preventing metabolic syndrome, preserving mental health, and improving sleep quality. Physical activity encompasses a wide range of exercises, from walking to intense sports, which contribute to cerebral blood flow, a healthy sympathetic system, and cellular brain regeneration. Neglecting physical activity can lead to various neurological diseases, affecting around one billion people worldwide. The abandonment of prevention at different levels worsens the situation, including negligence of key factors, lack of preventive programs, and inadequate health care systems.^1^ This correspondence focuses on the role of physical activity interventions in preventing neurological diseases.

## 2 UNDERSTANDING THE LINK BETWEEN PHYSICAL ACTIVITY AND BRAIN HEALTH

The brain is a complex organ responsible for various functions, including somatic motor and sensory control, perception, memory, learning, and emotions. To maintain optimal brain health, it is essential to focus on physical activity, prevention of metabolic syndrome, preservation of mental health, and improvement of sleep quality. There are seven key factors that contribute to brain health, including maintaining sufficient cerebral blood flow, an active sympathetic system, brain generation at the cellular level, a strong immune system, proper oxygen saturation, angiogenesis for delivering oxygen and nutrients, and hormonal balance.^2^

By following these preventive measures, it is possible to reduce the risk of neurological diseases such as ischemic stroke, hypoxic ischemia encephalopathy, neurodegenerative diseases, brain infections, psychiatric disorders, and cognitive decline caused by stress hormones. Neglecting physical activity and preventive measures can lead to a rise in neurological diseases worldwide, as reported by the WHO.^2^

**Figure 1:**
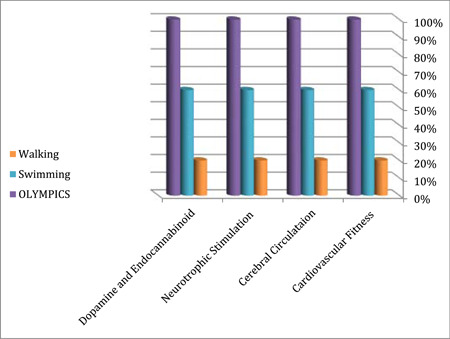
Illustrates the potential mechanisms linking between physical activity and brain health in which each potential mechanism (x‐axis) is stimulated differently according to the intensity of physical activity (colored bars) corresponding to the suitable percentage of brain health achievement (y‐axis).

Physical activity directly influences brain health through various mechanisms, including improving cardiovascular fitness, enhancing cerebral circulation, stimulating neurotrophic factors like brain derived neurotrophic factor (BDNF) for neuron protection and neurogenesis, and increasing dopamine production, which impacts movement, memory, pleasure, and satisfaction. By understanding the significance of physical activity interventions, we can explore their role in preventing neurological diseases effectively (Figure 1).

## 3 BENEFITS OF PHYSICAL ACTIVITY FOR BRAIN HEALTH

### 3.1 Overview of the positive effects of physical activity on the brain and nervous system

Pathomechanisms that lead to neurological diseases involves environmental, behavioral, genetic as well as lifestyles related factors. Among many we can state decreased neuroplasticity, oxidative stress, protein misfolding and neuroinflammation. Not only Physical activity can have a favorable impact on various disorders but also can mitigate inflammation, enhance antioxidant defenses and encourage the development of neuroprotective proteins.

Physical activity has different positive effects on the brain and nervous system. Physical activity works by preventing neurodegeneration and improves synaptic connections and neuronal regeneration. It can improve memory and reduce anxiety or depression Additionally, it can improve cognitive abilities, aid in memory and concentration improvement, and lift mood and mental well‐being As a result, physical exercise can lower your risk of cognitive decline and dementia. Stroke, AD, and PD risk may be elevated by a lifestyle lacking enough exercise training. Aerobic exercise improved cognitive function in older persons.^3^

### 3.2 Discussion of the neuro‐protective properties of physical activity

Brain insulin signaling can both prevent and correct abnormalities in BDNF transport, which are necessary for neuronal survival and the maintenance of vital brain functions. Diabetes, obesity, cardiovascular disease, hypertension, and all have links to insulin dysregulation. Learning and memory problems aberrant brain insulin signaling pathways are related to different neurodegenerative disorders.^3^

### 3.3 Examination of the cognitive benefits of physical activity and its role in neuroplasticity

**Figure 2:**
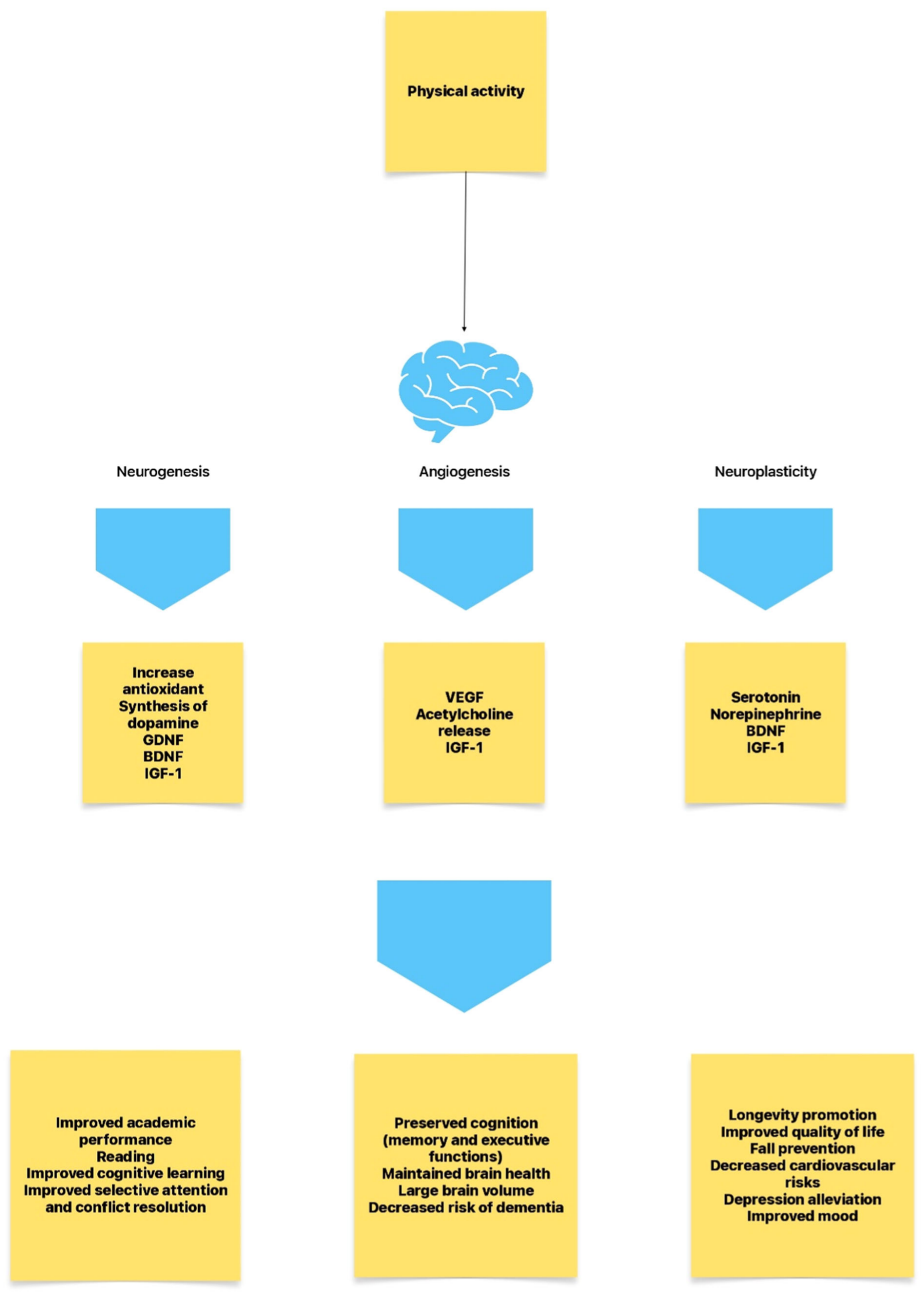
Physical activity stimulating Neurogenesis, Angiogenesis and Neuroplasticity and its benefits illustrated via respective pathophysiologies.

The human brain adapts to changing demands by altering its functional and structural properties (“neuroplasticity”) which results in learning and acquiring skills activity improves cognitive functions such as attention, memory, executive functions (Converging evidence from research on both humans and animals points to the facilitation of cognitive functions through the neuroplasticity of specific brain areas during physical activity. In the hippocampus and prefrontal cortex, it encourages the growth of new neurons and synapses, strengthens neuronal connections, and increases sanative plasticity. The enhancement of learning and memory functions via neuroplastic opportunities is known to boost cognitive performance as well as a person's resistance to age‐related cognitive decline^4^ (Figure 2).

## 4 TYPES OF PHYSICAL ACTIVITY INTERVENTIONS

### 4.1 Overview of various types of physical activity interventions

Various types of physical activity interventions include sit‐stand desks, aerobics exercises, introduction of walking during breaks, cycling, reducing sitting time, jogging which improve cardiovascular fitness. (Resistance training, such as bodyweight exercises, weightlifting helps in muscular strengthening and endurance. Exercises that involves flexibility such as yoga or stretching positively affects range of motion.^3^

### 4.2 Discussion of the duration, frequency, and intensity of physical activity required for optimal brain health

After a session of moderate‐to‐vigorous exercise, certain advantages of physical activity on brain health begin to materialize. Adults should engage in 150 min of moderate activity or 75 min of intense activity per week for the best outcomes. The most recent Physical Activity Guidelines for Americans recommend that adults exercise for 150 min a week at a moderate intensity and two days a week to build muscle. Numerous studies have shown that exercise can delay the onset of degenerative diseases including Alzheimer's, diabetes, and multiple sclerosis as well as heal, to some extent, the harmful effects of a sedentary lifestyle. The effects of exercise are analgesic, antidepressant, and even mood‐enhancing. It also improves memory and cognitive performance.^3^, ^4^

## 5 RECOMMENDATIONS AND PRACTICAL IMPLICATIONS

Physical activity recommendations are essential for people of all ages and abilities, including those with neurological diseases or conditions. While neurological diseases can present unique challenges, engaging in regular physical activity can have significant benefits for managing symptoms, improving overall health, and enhancing quality of life. Here are some comprehensive recommendations and strategies tailored for individuals with neurological conditions.^5^, ^6^, ^7^

### 5.1 Consult with health care professionals

Before starting any physical activity program, individuals with neurological diseases should consult with their health care provider, especially if they have specific health concerns or limitations. Health care professionals can help tailor exercise plans to meet individual needs and ensure safety.

### 5.2 Adapted exercise programs

Individuals with neurological conditions often require specialized exercise programs tailored to their abilities and limitations. Physical therapists, occupational therapists, and other health care professionals with expertize in neurological rehabilitation can design customized exercise plans to address specific challenges and goals.

### 5.3 Multidisciplinary approach

Managing neurological diseases may require a multidisciplinary approach, involving various health care professionals such as neurologists, physical therapists, speech therapists, and occupational therapists. Collaborative efforts can help address different aspects of the condition and enhance overall care.

### 5.4 Physical activity types

Physical activity for individuals with neurological diseases can include a combination of aerobic exercises, strength training, flexibility exercises, and balance and coordination activities. These exercises can help improve mobility, reduce spasticity, increase muscle strength, and promote overall well‐being.

### 5.5 Progressive approach

Starting with low‐impact activities and gradually increasing intensity and duration is important for those with neurological conditions. A progressive approach allows the body to adapt, minimizes the risk of injury, and increases the likelihood of maintaining a consistent exercise routine.

### 5.6 Incorporate assistive devices

For individuals with mobility limitations, the use of assistive devices such as canes, walkers, or adaptive equipment can enable safe and effective participation in physical activities.

### 5.7 Accessible facilities and programs

Ensuring that recreational facilities and exercise programs are accessible to individuals with neurological conditions is crucial. This includes providing ramps, accessible parking, adapted exercise equipment, and trained staff who understand the needs of this special population.

### 5.8 Group exercise and social support

Participating in group exercise classes or support groups can provide social engagement, motivation, and a sense of community for individuals with neurological conditions. It can also help individuals overcome feelings of embarrassment or isolation.

### 5.9 Consistency and motivation

Encouraging individuals with neurological diseases to remain consistent with their physical activity routine and providing positive reinforcement and motivation can help them stay committed to their exercise goals.

### 5.10 Monitor progress

Regularly monitoring physical activity progress and functional improvements can help individuals track their achievements and adjust their exercise programs accordingly.

Physical activity is a powerful tool for managing the effects of neurological diseases. With the guidance of health care professionals and tailored exercise programs, individuals with neurological conditions can experience improved physical function, increased independence, and enhanced overall well‐being.^6^, ^7^

## 6 CONCLUSION: FUTURE DIRECTIVES

This correspondance have emphasized the significant impact of physical activity as a method to maintain brain activity and health especially when it comes to age‐related neurodegenerative disease, such as Alzheimer's and Parkinson's disease. In addition, with the surge of new Neuroimaging methods, researchers have shifted away from the usage of treatments to a “lifestyle” based approach that comprehends many factors, including diet, exercise, employment, cognitive activities and socialization.^8^ Even though the mechanism by which physical activity improves brain health are yet to be more determined, studies highlighted a number of mechanisms in which exercise may modulate brain health, this involves an influence on Alzheimer's disease pathology (decrease deposition of Aβ and hyperphosphorylated tau), growth factors release (neutrophils), regulation of hormonal levels along with an improvement in brain volume and functional health. We should also note that the reduction of cardiovascular diseases and metabolic disorders have a positive activity on the brain's general aspect as well.^9^, ^10^ Nevertheless, no single superior method of training has been established; therefore aerobic, strength training or a combination of both could be an excellent start yet to be individualized based on future genetic studies.

## AUTHOR CONTRIBUTIONS

**Burhan Kantawala**: Writing—review and editing. **Nagham Ramadan**: Writing—original draft; Writing—review and editing. **Youmna Hassan**: Writing—original draft; Writing—review and editing. **Violette Fawaz**: Writing—original draft; Writing—review and editing. **Nadine Mugisha**: Writing—original draft; Writing—review and editing. **Abubakar Nazir**: Supervision; Visualization; Writing—original draft; Writing—review and editing. **Magda Wojtara**: Writing—review and editing. **Olivier Uwishema**: Writing—review and editing.

## CONFLICT OF INTEREST STATEMENT

The author declare no conflict of interest.

## TRANSPARENCY STATEMENT

The lead author Abubakar Nazir affirms that this manuscript is an honest, accurate, and transparent account of the study being reported; that no important aspects of the study have been omitted; and that any discrepancies from the study as planned (and, if relevant, registered) have been explained.

## Data Availability

Not Applicable.
